# Reduced sphingosine kinase-1 and enhanced sphingosine 1-phosphate lyase expression demonstrate deregulated sphingosine 1-phosphate signaling in Alzheimer’s disease

**DOI:** 10.1186/2051-5960-2-12

**Published:** 2014-01-27

**Authors:** Johnatan Ceccom, Najat Loukh, Valérie Lauwers-Cances, Christian Touriol, Yvan Nicaise, Catherine Gentil, Emmanuelle Uro-Coste, Stuart Pitson, Claude Alain Maurage, Charles Duyckaerts, Olivier Cuvillier, Marie-Bernadette Delisle

**Affiliations:** 1 CHU Toulouse, Service d’Anatomie Pathologique, 31059 Toulouse, France; 2 Université de Toulouse III, Faculté de Médecine Rangueil, 31062 Toulouse, France; 3 CNRS, Institut de Pharmacologie et de Biologie Structurale, Toulouse, France; 4 Université de Toulouse, UPS, IPBS, Toulouse, France; 5 Service d’épidémiologie, CHU Toulouse, 31059 Toulouse, France; 6 Inserm, UMR 1037, 31432 Toulouse, France; 7 Centre for Cancer Biology, SA Pathology, Frome Road, Adelaide, SA 5000, Australia; 8 Inserm, UMR 837, 59045 Lille, France; 9 CHU Lille, Service de Pathologie, 59037 Lille, France; 10 Université Lille Nord de France, 59000 Lille, France; 11 Laboratoire de Neuropathologie et Centre de Recherche de l’ICM, Hôpital de la Salpétrière, 75013 Paris, France

**Keywords:** Alzheimer’s disease, Sphingolipids, Beta amyloid, Sphingosine kinase-1, Sphingosine 1-phosphate lyase, Neuropathology

## Abstract

**Background:**

The accumulation of beta amyloid (Aβ) peptides, a hallmark of Alzheimer’s disease (AD) is related to mechanisms leading to neurodegeneration. Among its pleiotropic cellular effects, Aβ accumulation has been associated with a deregulation of sphingolipid metabolism. Sphingosine 1-phosphate (S1P) derived from sphingosine is emerging as a critical lipid mediator regulating various biological activities including cell proliferation, survival, migration, inflammation, or angiogenesis. S1P tissue level is low and kept under control through equilibrium between its synthesis mostly governed by sphingosine kinase-1 (SphK1) and its degradation by sphingosine 1-phosphate lyase (SPL). We have previously reported that Aβ peptides were able to decrease the activity of SphK1 in cell culture models, an effect that could be blocked by the prosurvival IGF-1/IGF-1R signaling.

**Results:**

Herein, we report for the first time the expression of both SphK1 and SPL by immunohistochemistry in frontal and entorhinal cortices from 56 human AD brains. Immunohistochemical analysis revealed a decreased expression of SphK1 and an increased expression of SPL both correlated to amyloid deposits in the entorhinal cortex. Otherwise, analysis of brain tissue extracts showed a decrease of SphK1 expression in AD brains whereas SPL expression was increased. The content of IGF-1R, an activator of SphK1, was found decreased in AD brains as well as S1P_1_, the major receptor for S1P.

**Conclusions:**

Collectively, these results highlight the importance of S1P in AD suggesting the existence of a global deregulation of S1P signaling in this disease from its synthesis by SphK1 and degradation by SPL to its signaling by the S1P_1_ receptor.

## Background

Alzheimer’s disease (AD) is a devastating neurodegenerative disorder which is characterized by two principal features: i) intracellular accumulation of hyperphosphorylated tau protein constituting neurofibrillary tangles (NFT) and neuropil threads; and ii) extracellular accumulation of β-amyloid (Aβ) peptide, major component of diffuse, focal and stellate deposits – the focal deposit constituting the core of the senile plaques [1]. These lesions are likely implicated in neuronal death and synaptic dysfunction associated with cerebral atrophy and cognitive impairment, characteristics of AD [2]. According to the stage of the disease, they can be confined to a specific location (i.e. entorhinal cortex, hippocampus) or be widely distributed in the brain [3-5]. Even if definite causes are not clearly identified, several molecular mechanisms have been involved in the pathogenesis of AD: mutations of APP or of presenilins, epsilon 4 allele of ApoE, excessive Aβ production and/or reduced removal, tau protein abnormalities, oxidative stress and lipid metabolism alteration [6-9].

**Figure 1:**
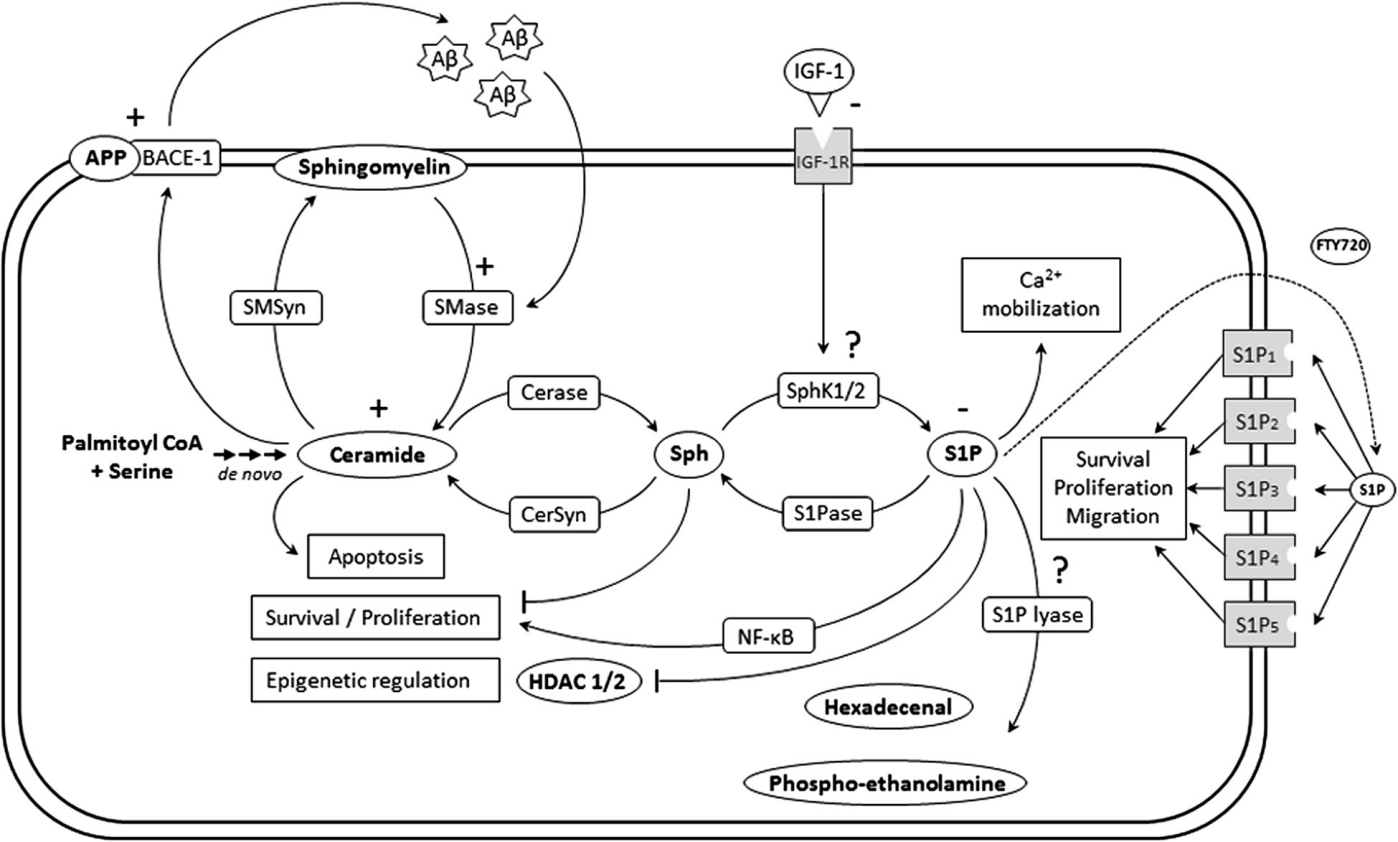
**Schematic representation of the sphingolipid rheostat in AD context.** Ceramide can be generated *de novo* or by hydrolysis of sphingomyelin by sphingomyelinase. Ceramide is subsequently metabolized by ceramidase to generate sphingosine which in turn produces sphingosine 1-phosphate through phosphorylation by sphingosine kinase-1 and sphingosine kinase-2. All these reactions are reversible. Sphingosine 1-phosphate can be catabolized into hexadecenal + phospho-ethanolamine by the action of sphingosine 1-phosphate lyase. Sphingosine 1-phosphate exerts intracellular signaling which can promote, calcium mobilization, epigenetic modulation and survival effects. S1P has also extracellular effects by signaling through 5 G protein-coupled receptors. The agonist FTY720 can bind S1P_1,3,4,5_. IGF-1/IGF-1R signaling pathway is able to activate SphK1, inducing its translocation to the plasma membrane. In AD, Aβ peptide is able to upregulate sphingomyelinase activity which consequently increases the level of ceramide. In turn, ceramide stabilizes BACE-1 which increases APP cleavage leading to generation of Aβ peptide [22]. S1P level as IGF-1R level are decreased in AD [23]. SMSyn, sphingomyelin synthase; SMase, sphingomyelinase; Cerase, ceramidase; CerSyn, ceramide synthase; Sph, sphingosine; SphK1/2, sphingosine kinase 1/2; S1Pase, sphingosine 1-phosphate phosphatase.

Sphingolipids are ubiquitous lipid components of membranes that are metabolized to form signaling molecules associated with cellular processes important for health and disease [10,11] (Figure 1). One of the most important of these metabolites is sphingosine 1-phosphate (S1P), which regulates pleiotropic biological activities such as proliferation, survival, migration, inflammation, or angiogenesis [10,11]. S1P is generated from sphingosine, the backbone component of all sphingolipids and a pro-apoptotic sphingolipid [12] in a reaction mainly catalyzed by the sphingosine kinase isoform 1, SphK1 [13]. In turn, SpkK1 can be activated by multiple stimuli as IGF-1 signaling [14]. The balance between the levels of S1P and its metabolic precursors ceramide and sphingosine has been regarded as a switch that could determine whether a cell proliferates or dies [15]. S1P can be secreted and signal as a ligand of five high-affinity G protein-coupled receptors (GPCR), named S1P_1-5_[16]. These receptors differ in their tissue distribution and the specific effect of S1P is driven by the predominance of the S1P receptor subtypes expressed [16]. Intracellular functions of S1P also exist with recent studies linking S1P to epigenetic regulation, calcium mobilization or activation of NF-κB [11,17,18]. Importantly, the agonist-induced S1P production as well as its downstream effects can be disrupted by inhibition of SphK1 gene expression or enzymatic activity illustrating that SphK1 plays a crucial role in the observed cellular effects played by S1P [19]. S1P can irreversibly be degraded into hexadecenal and ethanolamine phosphate by sphingosine 1-phosphate lyase (SPL) [20]. Interestingly, recent clinical observations have suggested an inverse relationship between SPL and SphK1 activities on the level of S1P in prostate cancer specimens implying that the overall increased S1P level commonly observed in cancer does not merely reflect overexpression of SphK1 activity, but could also be a consequence of loss of SPL expression [21].

In the brain, alteration of sphingolipid metabolism is believed to be essential for neuronal function as evidenced in a number of severe disorders besides AD including Niemann Pick disease, amyotrophic lateral sclerosis, Parkinson and AIDS dementia [24]. With regard to AD, most of the post-mortem studies have examined the level of ceramide and sphingosine, the pro-apoptotic precursors of sphingosine 1-phosphate, or enzymes responsible for their generation such as acid sphingomyelinase (which degrades sphingomyelin to ceramide) or acid ceramidase (which converts ceramide to sphingosine). For instance, acid sphingomyelinase activity and ceramide content are increased in the frontotemporal area [25-28]. A positive correlation was found between acid sphingomyelinase activity and Aβ or phosphorylated tau in this region, suggesting that increased ceramide levels are associated with AD pathology [24]. The involvement of sphingosine is unclear with either increased [25,29] or decreased [22,26] content in AD as compared to normal brains. On the contrary, one clinical study has reported a decrease in S1P expression in AD tissues [22]. Interestingly, this decrease of S1P level was negatively correlated with Aβ and phosphorylated tau protein levels [22]. Moreover, ceramide level has been reported to be increased in CSF from patients with moderate severity of AD [30] whereas low levels of plasmatic ceramide have been reported in the early stages of memory impairment [31,32]. Thus, the levels of these sphingolipids might be related to disease stage and represent an interesting pool of biomarkers for AD.

In cell culture models, a wealth of studies have firmly established the deleterious effect of ceramide on glial and neuronal cells exposed to Aβ peptides [26,33,34]. In addition to mediate the pro-apoptotic effect of Aβ, ceramide can also promote Aβ biogenesis by activating and stabilizing BACE-1 [35]. Conversely, S1P protects neuronal cells from apoptosis [36] notably in response to Aβ peptides [33]. Moreover, SphK1/S1P signaling was found to be a major transducer of two important growth factors, IGF-I and TGF-β1, whose neuroprotective effects against Aβ are well recognized [33,37-39]. With regard to the S1P receptors S1P_1-5_[11], their contribution to AD has not yet been investigated. However, FTY720, an agonist of S1P_1,3,4,5_, developed as an immunomodulatory drug and currently prescribed for multiple sclerosis, is able to restore passive avoidance memory in a rat model of AD as efficiently as Memantine, a finding supporting the existence of a direct action of this drug on neurons through S1P receptors [40].

Herein, we report for the first time the expression of SphK1 and SPL, the two main enzymes controlling the level of S1P, in frontal and entorhinal cortices of brains from AD patients, and their interaction with Aβ deposits distribution in the cortical layers. The expression of SphK1 and SPL was also assessed by western blot on brain tissue extracts together with SphK2, the minor isoform of sphingosine kinase, and S1P_1_, the most important S1P receptor and IGF-1R, whose activation promotes activation of SphK1 and production of S1P.

## Methods

### Human brain tissues

Human brain tissues were provided by certified French biological resource centers from Lille (DC-2008-642), Paris (DC-2009-957) and Toulouse (AC-2009-973), and by the national brain bank GIE Neuro-CEB (AC-2007-5). This study is compliant with the Helsinki Declaration and has been approved by CPP (Comité de protection des personnes du Sud-Ouest et Outre-Mer I) ethical committee (PHRC 1802, #1-09-54). Post-mortem tissues from 56 AD patients were included in the imunohistochemical study (Table 1). Once extracted, hemi-brains were fixed with formalin (4% in PBS) during approximately 1 month. Samples from frontal and entorhinal cortices were embedded in paraffin. These blocks were cut serially to obtain 4 μM sections. The diagnosis of AD was made according to current criteria of NIA – Alzheimer’s Association [41]. The assessment included Braak and Thal staging. For immunoblots, fresh samples (independent from those used for immunohistochemical study) from frontal cortex and hippocampus of 4 AD cases and 3 non demented age-mached controls were used (Table 2).

**Table 1:** Characteristics of patients included in the immunohistochemistry study

**Variable**	**AD patients (N=56)**
Age at death (years)	
Median [IQR]	80.0 [72.0-87.0]
Range	56.0, 96.0
Sex – n of patients (%)	
Female	37 (66.1)
Male	19 (33.9)
Post mortem interval	
Median [IQR]	26.5 [19.0-34.0]
Range	3.0, 96.0
Missing (n)	4
Braak Stage – n of patients (%)	
IV	1 (1.8)
V-VI	55 (98.2)
Thal – n of patients (%)	
2	2 (3.57)
3	5 (8.93)
4	16 (28.57)
5	33 (58.93)

**Table 2:** Characteristics of patients included in the immunoblot study

**Variable**	**Controls (N = 3)**	**AD patients (N = 4)**
Age at death (years)		
Median [IQR]	73.0 [69.0-92.0]	73.0 [72.0-73.0]
Range	69.0-92.0	72.0, 89.0
Sex – n of patients (%)		
Female	1 (33.3)	2 (50.0)
Male	2 (66.6)	2 (50.0)
Post mortem interval		
Median [IQR]	36.5 [10.0-63.0]	56.5 [44.0-59.0]
Range	10.0-63.0	44.0, 59.0
Missing (n)	1	
Braak Stage – n of patients (%)		
0	2 (66.6)	
II	1 (33.3)	
VI		4 (100.0)
Thal – n of patients (%)		
0	3 (100.0)	
4		1 (25.0)
5		3 (75.0)

### Immunohistochemistry

Paraffin-embedded, formalin-fixed sections were deparaffinized in xylene, rehydrated in graded ethanol and washed for 5 min with Tris buffer saline (pH 7.6). Antigen retrieval was performed by immersing sections in boiling EDTA buffer (pH 9.0). Endogenous peroxidase and alkaline phosphatase were blocked by incubation of the sections for 10 min in Dual Endogenous Enzyme Block (Dako, Denmark). Double staining was carried out to evaluate the expression of SphK1 and the density of amyloid deposits (n = 56). Double staining was also carried out to evaluate the expression of SPL together with the density of amyloid deposits in 10 cases randomly selected. Sections were initially incubated with primary antibody directed against amyloid beta (mouse clone 6 F/3D, 1:100, Dako) during 2 hours at room temperature (RT). Sections were washed twice during 7 minutes in Tris-buffered NaCl solution with Tween 20 (pH 7.6, Dako). Immunostaining was revealed using BrightVision poly HRP-Anti-Mouse IgG (Immunologic, Netherland) during 30 min at RT and treated with diaminobenzidine/hydrogen peroxide (DAB) for 10 minutes (DAB + Liquid, Dako). Sections were rinsed for 5 minutes in tap water and then rinsed rapidly in distillated water. After this first step, sections were washed for 10 minutes with Tris buffer saline (pH 7.6) before incubation with primary antibody directed against SphK1 (rabbit polyclonal, 1:500, [21,42]) or SPL (rabbit polyclonal #HPA023086, 1:250, Sigma Aldrich) overnight at 4°C. The sections were washed twice during 7 minutes in Tris-buffered NaCl solution with Tween 20 (pH 7.6, Dako). Immunostaining was exposed using BrightVision poly AP-Anti-Rabbit IgG (Immunologic, Netherland) during 30 minutes at RT and treated with Liquid Fast Red (Abcam) for 30 minutes. Sections were counterstained with hematoxylin in alcohol solution. Slides were then mounted in Faramount Aqueous Mounting Medium (Dako).

### Qualitative and quantitative examination

Once mounted, slides were scanned with a digital scanner NanoZoomer (HAMAMATSU, Réf: 2.0-RS: C10730-13) to obtain high resolution virtual slides. Digitalized slides were analyzed with NDP View 2.0 software (HAMAMATSU). Morphometric investigation was carried out by two observers (M. B. Delisle & J. Ceccom) to determine the numerical density of amyloid deposits and of neurons expressing SphK1 or SPL at two levels: negative or mild (+) and strong (++) among the different cortical layers. Columns constituted of contiguous microscopic fields, from the pial surface to the white matter were drawn on each slide. As the fields were examined at a magnification of x400, each field was 300 μM × 150 μM in size. As the thickness of the cortex appeared to be variable between the different sections, after the counting step, the columns were standardized to 10 fields [43]. Field 1 corresponded to the cortex immediately under the pial surface and field 10 reached the white matter (Additional file 1). In each field, the number of profiles of Aβ deposits, of neurons and of neurons expressing low level (+) and high level (++) of SphK1 and of SPL was counted and reported on a database. For Aβ deposits, focal and diffuse plaques were recorded separately according to published discriminating features [1].

### Preparation of human brain homogenates and Western blotting

Frozen tissue samples were pulverized with “Mikro-Dismembrator” (Sartorius, France) and resuspended in lysis SDS sample buffer (50 mM Tris-HCl pH 6.8, 5% SDS, 10% glycerol and Complete EDTA-free protease inhibitor cocktail (Roche)). Samples were sonicated at 4°C then centrifuged at 13,000 × *g* for 10 minutes. Total protein concentration was assessed on the supernatant with the BCA Protein Assay (Interchim). Samples were prepared for electrophoresis by adding 5% β-mercapto-ethanol, 0.05% bromophenol blue and heating at 98°C for 3 minutes. Sixty μg of total proteins were loaded into each lane of a 10% polyarcrylamide gel and electrophoresed at 50 V in a MiniProtean Tetra System (Bio-Rad Laboratories, Irvine, CA, USA). After migration and 10 min of transfer with the Transblot Turbo (Bio-Rad Laboratories), nitrocellulose membranes were blocked with 4% skimmed milk, and washed 3 times with Tris-buffered saline buffer containing 0,05% Tween-20 (TBST). Blots were probed with either SphK1 (rabbit polyclonal #71700, 1:1000, Abcam), SphK2 (rabbit polyclonal #37977, 1:1000, Abcam), SPL (rabbit polyclonal #HPA023086, 1:2,000, Sigma Aldrich), S1P1 (rabbit clone EPR4538(2) #NBP1-95120, 1:5,000, Novus) and IGF-1R (mouse clone 3G5C1 #MAB10693, 1:2,000, Abnova) antibodies. After an overnight incubation at 4°C, the membranes were washed with TBST, labeled with a peroxidase-conjugated anti-rabbit or anti-mouse (dilution 1:3,000) secondary antibody (Clontech) and revealed by chemiluminescence (Clarity kit, Bio Rad). The density of the band of β–actin (anti-β-actin monoclonal antibody, AC-15, Sigma-Aldrich, 1:10,000) was used to normalize the signals.

### Data analysis

Statistical analysis was carried out with a multilevel linear mixed model to take into account non independent data (eight fields into two cerebral areas per subject were studied). Due to the poor representativeness of fields 1 non tissular zone and pial surface) and 10 (proximal white matter), they were not included in statistical analysis. As a strong relationship between the number of neurons and SphK1 expression was guaranteed because of mathematical coupling (ie the number of neuron expressing SphK1 in a specific field depends on the number of neurons observed in this same field), the relation between total number of neurons and SphK1 expression was estimated using the method of Oldham [44]. Correlations were estimated as significant at p < 0.05. The analysis was performed using “Stata 11.2 Statistical Software” (Stata Corporation, College Station, TX, USA).

## Results

### Immunohistochemical study

**Figure 2:**
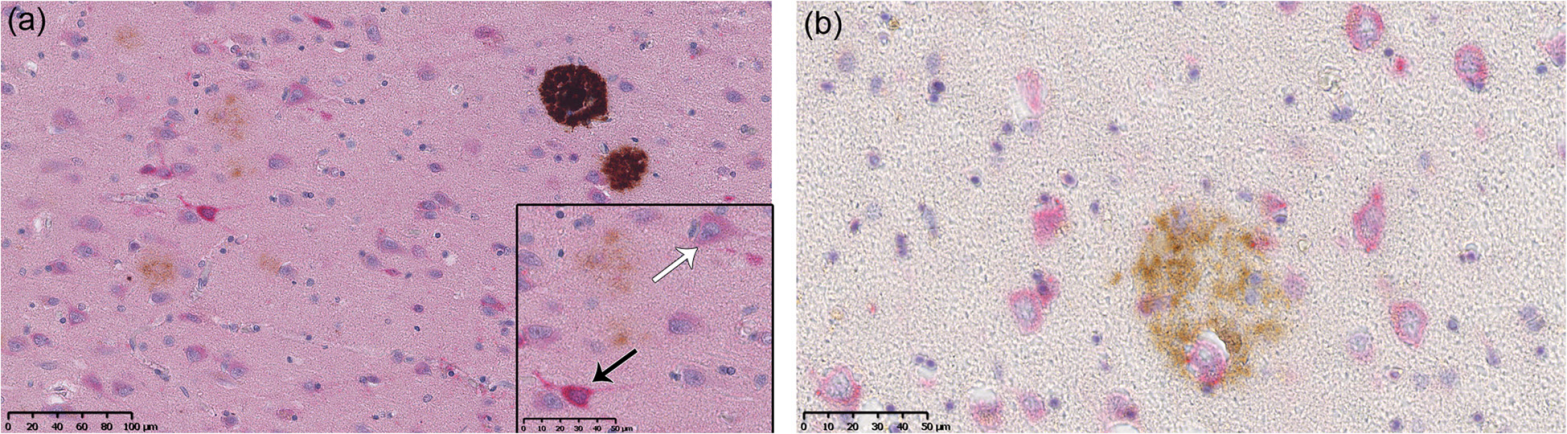
**Immunohistochemical study of SphK1/SPL expression and Aβ deposits. (a)** Double labeling of SphK1 and Aβ in AD brain section from entorhinal cortex. Diffuse and focal deposits are seen in different areas. X200. Inset: Strongly stained neurons (SphK1++, black arrow) and mild stained neurons (SphK1+, white arrow) are clearly distinguishable. X400 **(b)** Double labeling of SPL and Aβ in AD brain section from entorhinal cortex. A senile plaque is mainly surrounded by strong labeled neurons (SLP++). X400.

Most of the subjects were staged Braak V-VI and Thal 4 to 5, therefore the packing density of neurofibrillary tangles and senile plaques was high (Table 1). Cortical thickness variability was noticed and could be related to atrophy which is a common feature in AD. Morphological study showed a distribution of cortical layers over the 10 fields standardized columns which was consistent with literature [1,43]. For instance, in frontal and entorhinal cortex, cortical layer I was principally found in fields 1 and 2, cortical layers II & III were mostly represented in fields 2 to 6, layer IV was confined in fields 6 to 8, and layers V & VI was found in fields 7 to 10. The distribution of neurons over cortical layers was different as expected and did not follow the same pattern in the two studied cortical regions. Namely, in frontal cortex, the number of neurons was constant over layers II to V whereas their packing density was more fluctuant in entorhinal cortex. The distribution of Aβ deposits was consistent with the laminar distribution previously reported [1,43], namely a higher numerical density in cortical layers II and III (data not shown). Moreover, in our cohort, focal deposits were more represented than diffuse ones in frontal (β = 1.7; p < 0.001) and entorhinal (β = 1.08; p < 0.001) cortices, which is consistent with advanced stage of AD. SphK1/SPL staining was mainly observed in neurons (Figure 2).

#### Correlation between density of neurons and Aβ deposits

The packing density of neurons and Aβ deposits were uncorrelated in the frontal cortex (β = 0.049; p = 0.67) and inversely correlated in the entorhinal cortex (β = -0.34; p = 0.019). This negative correlation was only related to the presence of focal deposits (β = -0.39; p = 0.021) while diffuse ones were not found to impact the density of neurons (β = -0.17; p = 0.645). These results indicate an overall effect of Aβ focal deposits on neuronal density solely observable in entorhinal cortex which is consistent with morphological observations characteristics of end stage AD patients.

#### Correlation between Aβ deposits and SphK1 expression in AD brain

**Figure 3:**
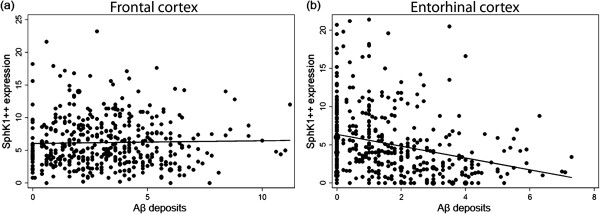
**Correlation between neurons with strong expression of SphK1 and Aβ deposits. (a)** Packing density of SphK1++ neurons and density of Aβ deposits are not correlated in frontal cortex. **(b)** A negative correlation between the packing density of SphK1++ neurons and the density of Aβ deposits is observed in entorhinal cortex. Data are represented as scatter plot with regression line.

The packing density of neurons in which SphK1 expression was high (SphK1++) was not correlated with Aβ deposits density in the frontal cortex (β = 0.04; p = 0.62) whereas it was inversely correlated in the entorhinal cortex (β = -0.77; p < 0.001) (Figure 3). This negative correlation was only related to the presence of focal deposits (β = -0.88; p < 0.001) while diffuse ones were not found to impact the density of neurons expressing Sphk1 at high level (β = -0.33; p = 0.218).

#### Correlation between Aβ deposits and SPL expression in AD brain

**Figure 4:**
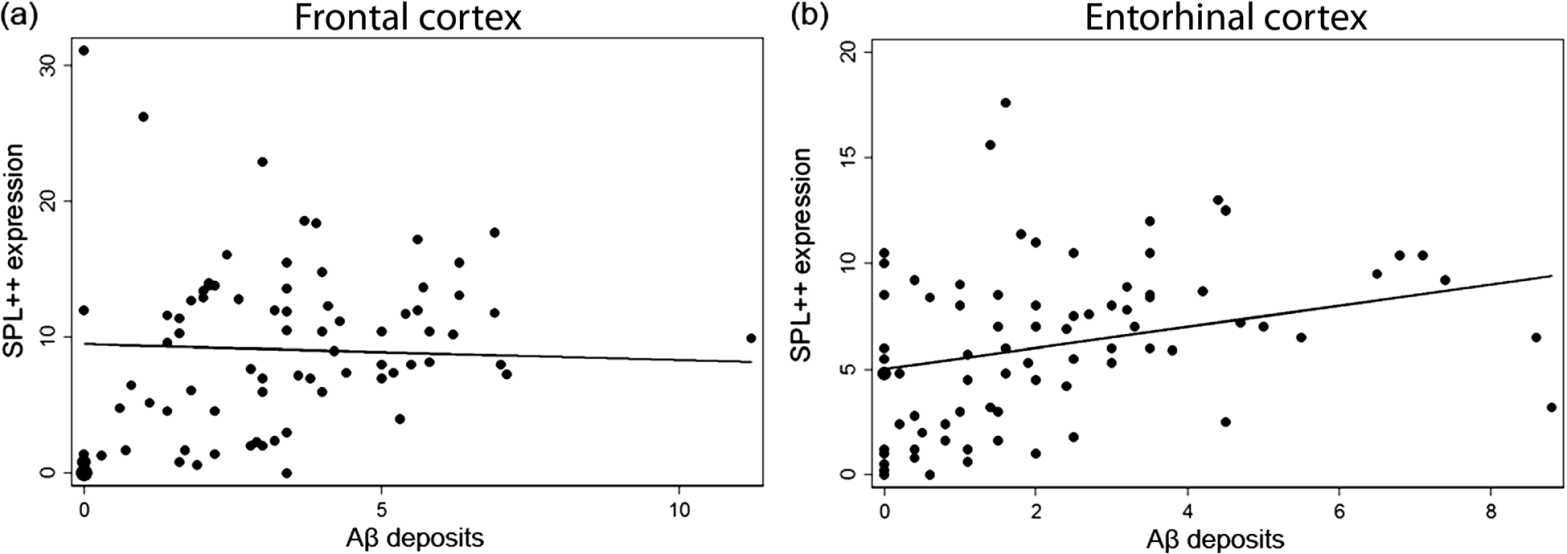
**Correlation between neurons with strong expression of SPL and Aβ deposits. (a)** Packing density of SPL++ neurons and density of Aβ deposits are not correlated in frontal cortex. **(b)** A positive correlation between the packing density of SPL++ neurons and the density of Aβ deposits is observed in entorhinal cortex. Data are represented as scatter plot with regression line.

The packing density of neurons with strong expression of SPL (SPL++) and the packing density of Aβ deposits were not correlated in the frontal cortex (β = -0.11; p = 0.72) whereas a significant correlation was found in the entorhinal cortex (β = 0.49; p = 0.014) (Figure 4). This positive correlation was only related to the presence of focal deposits (β = 0.85; p = 0.004) while diffuse ones were not found to impact the density of neurons expressing SPL at high level (β = - 0.47; p = 0.43).

#### Correlation between SphK1 expression and total neurons in AD brain

Statistical analysis revealed that SphK1 expression and total density of neurons were correlated in frontal cortex (β = 0.54; p < 0.001) and entorhinal cortex (β = 0.27; p < 0.001).

### Immunoblot analysis

**Figure 5:**
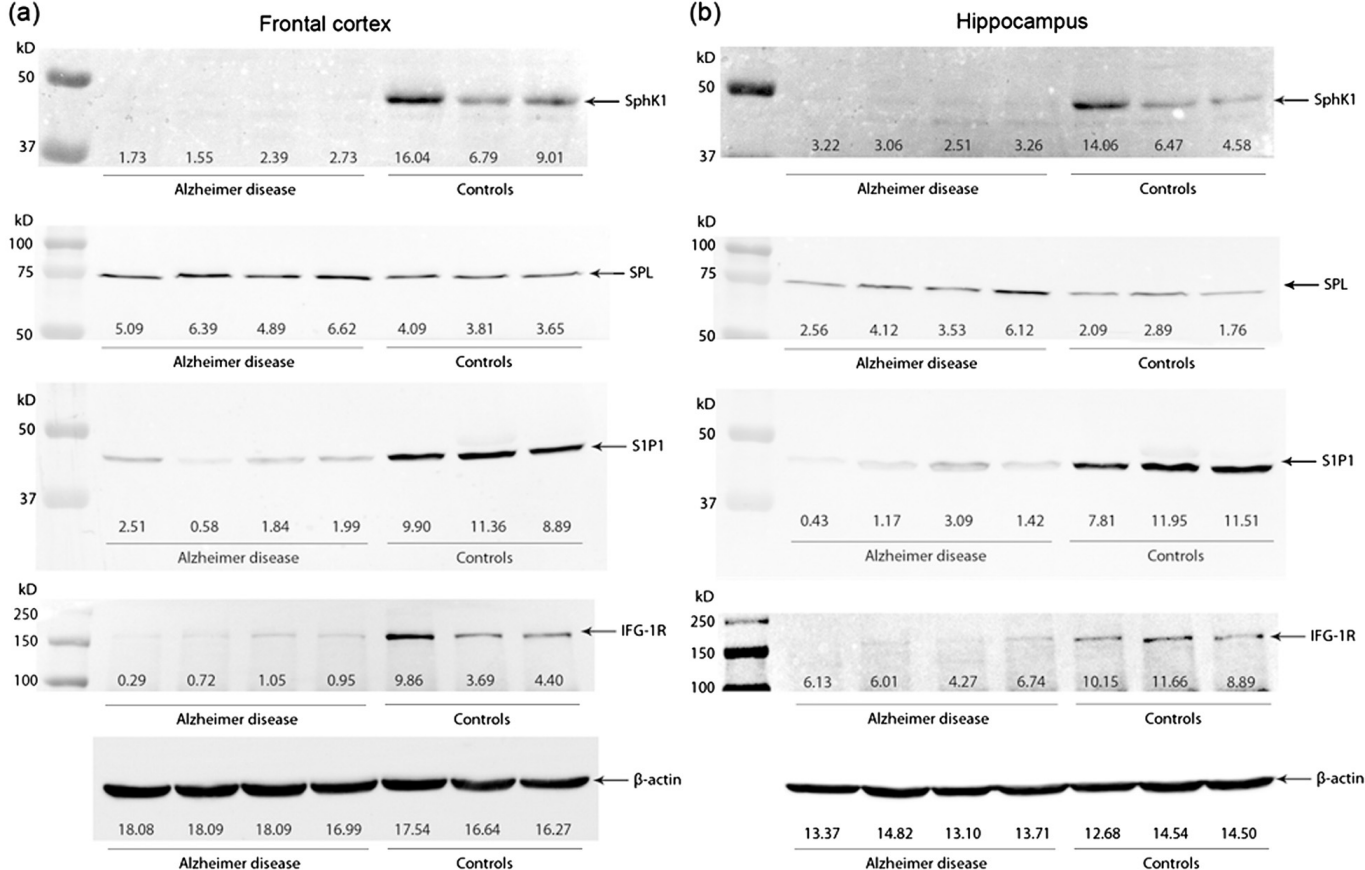
**Immunoblot analysis of SphK1, SPL, S1P**_**1 **_**and IGF-1R.** Tissue lysates from frontal cortex **(a)** and temporal cortex in the hippocampal area **(b)** of AD and control brains were assayed for SphK1, SPL, S1P_1_ and IGF-1R expression. Equal loading of protein was monitored using antibody to β-actin. The intensity of each band was quantified using ImageJ software and is provided as representative under corresponding blot. Values are expressed at 1/1000.

Each tissue lysate from frontal cortex and temporal cortex in the hippocampal area of AD and control brains (Table 2) was prepared to quantify the amount of SphK1 and SPL protein. In line with the immunohistochemistry analysis, there was a marked decrease in SphK1 content in AD extracts as compared to control (Figure 5). On the contrary, SPL expression was higher in AD extracts as compared to control especially in entorhinal cortex (Figure 5). We next assessed the level of SphK2, the other sphingosine kinase isoform but its expression was not different between AD and control samples (data not shown). Importantly, the expression of the S1P_1_ receptor, which notably mediates cell survival in response to S1P in various cell systems and whose expression is ubiquitous, was reduced in frontal and entorhinal cortex (Figure 5). Finally, a marked decrease in IGF-R1 expression was observed in AD samples (Figure 5).

## Discussion

While cancers are associated with alterations of cellular cycle inducing anarchic proliferation, neurodegenerative diseases are on the contrary associated with a cellular deregulation leading to neuronal death. As recently shown in a cohort study, elderly people suffering from cancer have a reduced risk of AD dementia and *vice versa*[45]. This observation underlines the existence of a relationship between these two major mechanisms of cellular function impairment. Interestingly, SphK1 overexpression leading to increase S1P signaling has been demonstrated to have an important role in cancer initiation, progression and resistance to therapeutics [13], whereas high levels of ceramide have been reported in AD brains [24]. Thus, in cancer and neurodegenerative diseases like AD, two opposite cellular fate outcomes could result from the imbalance of ceramide/S1P biostat [15]. Recently, Brizuela and coworkers reported that SphK1 expression was upregulated whereas SPL expression was downregulated in prostatic cancer. This original result showed that abnormal S1P level in prostatic malignant cells was not only related to overproduction by SphK1 but also to an important impairment of the elimination pathway provided by SPL [21]. In our study we reported the opposite situation, and showed for the first time that in AD, SphK1 expression was downregulated whereas SPL expression was upregulated. As a consequence of this deregulation, S1P levels should be decreased in cells and drive them to neurodegenerative processes.

In 2010, He and coworkers provided crucial information about the levels of ceramide and S1P in AD brains and assessed the expression level of enzymes implicated in ceramide/S1P metabolism but not SphK1 nor SPL [22]. The authors showed that Aβ was able to interact with sphingomyelinase and could induce *in fine* a decrease of S1P level. On the other hand, *in vitro* studies showed that Aβ, under oligomeric or fibrillary form, could trigger ceramide mediated apoptosis [22,46]. The lack of information about SphK1 and SPL in AD and their direct involvement in S1P metabolism led us to investigate their expression within AD brains and to assess their possible relationship with Aβ deposits which represent one of the principal hallmarks of this disease.

Western blot analysis showed that SphK1 expression was reduced in AD brains compared to non demented controls. This observation supports the idea that neuropathologic processes related to AD and especially Aβ accumulation may induce deleterious effects on the expression of principal actors of the sphingosine 1-phosphate metabolism. SphK2 which is largely less implicated in the overall production of S1P than SphK1 did not show any particular modification of its expression in AD brains which is consistent with literature [47]. Morphologically, SphK1 expression was dramatically decreased within neurons populating fields in which the density of Aβ deposit was the highest. These fields corresponded predominately to cortical layers II, III where neuritic plaques are preferentially found [43] and extended to layer IV. This result was significant for neurons from entorhinal cortex that are very vulnerable [48], whereas neurons from frontal cortex seemed to be more resilient to Aβ toxicity. However, the packing density of total neurons in frontal and entorhinal cortices was correlated with the packing density of neurons with high expression of SphK1. As SphK1 expression is related to survival effects, its downregulation in AD could induce an opposite outcome. We previously showed that SphK1 activity was also reduced when cultured cells were exposed to fibrillary Aβ 25-35 [33]. All these results tend to demonstrate that Aβ deposits are directly involved in the reduction of S1P production by modulating the expression and the activity of SphK1 and could eventually shift the death/survival balance in favor of neurodegenerative processes.

Inversely, SPL which is the final enzyme in the sphingolipid degradative pathway controls the only exit point for sphingolipid intermediates. In addition to regulating S1P levels, SPL is a gatekeeper of a critical node of lipid metabolic flow [20]. In our study, Western Blot examination of SPL expression showed a higher level of this enzyme in AD brains compared to controls. This observation suggests that SPL could be highly deregulated in AD and is consistent with literature that reported upregulation of SPL mRNA expression in AD brains correlated to progression of dementia [28]. Our immunohistological study on ten AD cases confirmed these data and provided complementary information. Aβ deposits packing density was not correlated with high expression of SPL within neurons from frontal cortex but was positively correlated with high expression of SPL within neurons from entorhinal cortex. Notably, SPL deficiency leads to resistance against apoptosis induced by chemotherapy or nutriment starvation [21,49]. In AD, two single nucleotide polymorphisms were detected in the *sgpl1* gene in late-onset AD, which suggests that variation in sgpl1 expression and/or function may confer susceptibility to late-onset AD [50]. Our data indicates that increase of SPL expression in AD could be one of the consequences of Aβ accumulation. Hexadecenal and phospho-ethanolamine produced by SPL from S1P degradation have been reported to induce apoptosis, among other effects [51]. As suggested by Aguilar and Saba in 2012, SPL upregulation may be involved in accumulation of hexadecenal which could induce neurological and cognitive defects in some pathologies as for example in Sjögren-Larsson syndrome. This hypothesis suggests an important involvement of SPL deregulation in the pathogenesis of AD and leads to consider this enzyme as a promising therapeutic target.

SphK1 activation is modulated by many agonists including IGF-1 which induces the translocation of SphK1 to the plasma membrane [52]. In a previous study, we showed that the deleterious effect of Aβ exposition on SphK1 activity could be reversed by adjunction of IGF-1 to the culture medium [33]. Here we show that IGF-1R expression is dramatically reduced in frontal and hippocampal regions of AD cases compared to controls. This result is consistent with literature [23] and introduces a possible candidate for mediating signaling between Aβ and SphK1. Post-mortem studies on AD brains showed that IGF-1 deficiency and resistance is related to the stage of the disease and then could be considered as causal in the pathogenesis of AD [23]. IGF-1R impairments lead to brain amyloidosis in rodents and IGF-1R confers to cells the ability to reduce exogenously applied oligomers [53,54]. This suggests that IGF-1R disorders are involved in Aβ accumulation and subsequent synaptic loss [55]. Here, we face a vicious circle in which Aβ induces a deregulation of IGF-1 signaling that in turn leads to overproduction of Aβ.

As S1P is able to trigger intracellular signaling pathways, it is also involved in an extracellular autocrine/paracrine signaling through five S1P receptors. Now well described, these receptors are involved in a wide range of signaling pathways including proliferation, survival, migration and cell-cell interactions [16]. Here we focused on S1P_1_ as it is the most represented in brain and its activation can lead to an increase of survival/prevention of apoptosis through PI3K and Akt signaling [56]. The important decrease of S1P_1_ expression in AD cases reported in our study could be related to a deregulation of S1P extracellular signaling induced by Aβ accumulation. This hypothesis is consistent with recent study which showed that FTY720, an agonist of S1P receptors with high affinity for S1P_1_ was able to reverse behavioral impairment in rat model of AD [40].

## Conclusion

In conclusion, our data extend previous *in vitro* findings regarding the effect of Aβ deposits on sphingolipid rheostat and show for the first time the decreased expression of SphK1 in AD brains. They provide the first evidence of increased expression of SPL related to amyloid deposits in a human neurodegenerative disease. Taken together, these original data reveal an imbalance in SphK1/SPL system which might play a crucial role in neurodegenerative disease. As cells are very sensitive to variations of S1P levels, the assessment of such variations in AD or early stages of memory impairment could be a promising prognostic tool as a biomarker in fluids.

### Consent

Written informed consent was obtained from the patient for the publication of this report and any accompanying images as required in the concerned authorized biological resource centers.

## Competing interests

The authors declare that they have no competing interests.

## Authors’ contributions

NL and YN carried out double labeling immunoassays and immunoblot experiments and participated in drafting the manuscript. CG and VLC performed statistical analysis and data interpretation. VLC participated in the conception of the study. CT carried out immunoblot experiment, analyzed data and participated in the conception of the study and drafting of the manuscript. SP provided SphK1 antibody and participated in drafting the manuscript. CAM, CD and EUC provided biological material and associated data for staging, participated in drafting and reviewing the manuscript. CD participated in the conception of the methodology used in the study. JC, MBD and OC conceptualized the design of the study, analyzed and interpreted data, and wrote the manuscript. JC managed the conduct of experiments. All authors read and approved the final manuscript.

## Supplementary Material

Additional file 1 **Virtual slides and counting method. (a)** Virtual slide obtained from hippocampal area section. The section is double labeled for SphK1 and Aβ. **(b)** Representative scale matrix designed on entorhinal cortex used for neurons and Aβ deposits counting. Boxes extend from pial surface to white matter. Click here for file
