# Supplementary figures and images for: Reduced sphingosine kinase-1 and enhanced sphingosine 1-phosphate lyase expression demonstrate deregulated sphingosine 1-phosphate signaling in Alzheimer’s disease
Source: Acta Neuropathol Commun. 2014 Jan 27;2:12. doi: 10.1186/2051-5960-2-12 (PMC3912487; doi:10.1186/2051-5960-2-12)

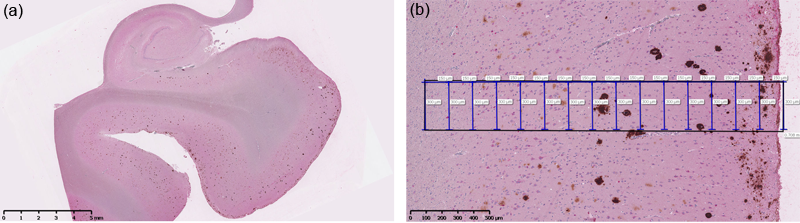

Supplement: Additional file 1 — Virtual slides and counting method. (a) Virtual slide obtained from hippocampal area section. The section is double labeled for SphK1 and Aβ. (b) Representative scale matrix designed on entorhinal cortex used for neurons and Aβ deposits counting. Boxes extend from pial surface to white matter. [file 2051-5960-2-12-S1.tiff]
